# Toward evidence-informed medical curricula: A scoping review protocol on social skills and emotional regulation

**DOI:** 10.1016/j.mex.2025.103161

**Published:** 2025-01-11

**Authors:** Thaysa Castro Molina, William Donegá Martinez, Vinicíus Araújo Pereira, Alba Regina de Abreu Lima, Vânia Maria Sabadoto Brienze, Emerson Roberto dos Santos, Valdir Carlos Severino Júnior, Camila Borge, Júlio César André

**Affiliations:** a Postgraduate Program in Health Sciences, Faculty of Medicine of São José do Rio Preto, São José do Rio Preto, Brazil; b Center for Studies and Development of Health Education, Faculty of Medicine of São José do Rio Preto, Brazil

**Keywords:** Social skills, Emotional regulation, Medical students, Medical education, Scoping review, Toward Evidence-Informed Medical Curricula: A Scoping Review Protocol on Social Skills and Emotional Regulation

## Abstract

Medical education is considered psychologically challenging, and medical students often report higher levels of psychological distress compared to non-medical students and the general population. This scoping review aims to identify and map the available scientific evidence on social skills and emotional regulation in medical students, characterize existing studies, identify knowledge gaps, and provide a synthesis of evidence to inform educational practice and curriculum development. This scoping review will follow the methodological frameworks proposed by the Joanna Briggs Institute (JBI) and the PRISMA-ScR checklist. Relevant studies will be searched in electronic databases (EMBASE, ERIC, PubMed, Science Direct, Scopus, and Web of Science) and gray literature sources. The search strategy will use descriptors in English, Portuguese, and Spanish, combined with Boolean operators. Two reviewers will independently screen titles, abstracts, and full-texts for inclusion. Data will be extracted using a standardized form and synthesized narratively. Qualitative and quantitative analyses, including thematic analysis and meta-analysis analysis (if appropriate), will be employed to provide a comprehensive overview of the evidence. The review will provide a comprehensive overview of the available scientific evidence on social skills and emotional regulation in medical students, identifying study characteristics, knowledge gaps, and areas for future research. The findings of this scoping review may inform educational practice and guide the development of strategies to promote the well-being and performance of medical students by addressing social skills and emotional regulation in medical curricula. The review protocol will be registered in the Open Science Framework (OSF).

## Graphical abstract

**Figure:**
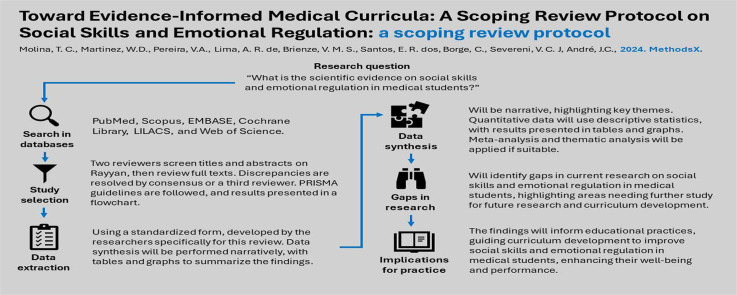

Specifications table

**Table:** 

Subject area:	Medicine and Dentistry
More specific subject area:	Medicine, methods of synthesis of knowledge, study designs
Name of your protocol:	Toward Evidence-Informed Medical Curricula: A Scoping Review Protocol on Social Skills and Emotional Regulation
Reagents/tools:	Not applicable
Experimental design:	Not applicable. This protocol describes a scoping review
Trial registration:	Not applicable. This scoping review is registered in the Open Science Framework (https://doi.org/10.17605/OSF.IO/XYW8N)
Ethics:	This study does not involve human beings or non-human animals because it is secondary research
Value of the Protocol:	Main scientific contribution: 1. Characterize existing studies on social skills and emotional regulation in medical students in terms of study design, population, interventions, and evaluated outcomes; 2. Identify gaps in current knowledge and areas for future research; 3. Provide a synthesis of available evidence to inform educational practice and the development of medical curricula that address social skills and emotional regulation.

## Background

Medical education is considered psychologically challenging, so it is not surprising that medical students report more psychological distress than non-medical students and the general population [[1], [2], [3], [4]].

The psychological well-being of medical students is crucial not only for their academic performance but also for their future roles as healthcare providers. Social skills and emotional regulation are key components of this well-being, yet they are often overlooked in traditional medical curricula [5].

Socioemotional skills, such as the ability to recognize, understand, and regulate one's own emotions and those of others, are associated with physical and emotional health. Programs aimed at training these skills are promising in improving competencies relevant to adaptive social and emotional functioning [6]. In the context of medical education, these skills can potentially mitigate stress, enhance resilience, and improve patient-doctor communication [[7], [8], [9]].

Considering that medical training inevitably involves experiences of stress, fear of failure, and expectations of overcoming, which require emotional regulation skills, it may be beneficial to include curricula that strengthen students' ability to focus and reflect on their own emotions and emotional regulation strategies [10,11]. However, the extent to which these skills are currently addressed in medical education, and the most effective methods for developing them, remain unclear.

Given the potential importance of social skills and emotional regulation in medical education [12,13], a comprehensive review of the existing literature is necessary. This scoping review aims to map the current landscape of research on these topics within medical education, identify gaps in knowledge, and inform future curriculum development and research directions.

## Description of protocol

This scoping review will be conducted according to the methodological frameworks proposed by the Joanna Briggs Institute (JBI) for scoping reviews [14]. The review process will follow these steps: (1) identification of the research question; (2) identification of relevant studies; (3) study selection; (4) data extraction; and (5) synthesis and reporting of results [15,16].

The PRISMA-ScR (PRISMA extension for Scoping Reviews) checklist will be used to ensure transparency and quality of reporting [17]. The review protocol will be registered in the Open Science Framework (OSF) to promote transparency and reproducibility of the review process [18]. It is worth noting that a search was conducted in databases and scientific reviews, identifying that there are no available reviews on this topic within the stipulated period for this study.

In addition to the main steps outlined above, this scoping review will employ several strategies to ensure a comprehensive and rigorous review process:

1. Comprehensive search strategy: A librarian specializing in digital search strategies will collaborate with the research team to develop and refine the search strategy [19]. This will ensure a thorough and sensitive search across multiple databases and gray literature sources [20].
2. Iterative screening process: The study selection process will be iterative, allowing for refinement of inclusion/exclusion criteria as the reviewers become more familiar with the literature. This approach is particularly suitable for scoping reviews, where the breadth of evidence may not be fully known at the outset [16].
3. Detailed data extraction: A comprehensive data extraction form will be developed to capture not only basic study characteristics but also nuanced information about social skills and emotional regulation interventions, measurement tools, and outcomes. This will allow for a more in-depth analysis of the evidence [21].
4. Mixed-methods synthesis: The review will employ both narrative [22] and quantitative synthesis methods, as appropriate. Thematic analysis will be used to identify recurring themes in qualitative data, while meta-analysis may be considered for combining quantitative results from similar studies [23].
5. Stakeholder consultation: To enhance the relevance and applicability of the review findings, we will consult with key stakeholders, including medical educators, students, and mental health professionals, during the final stages of the review. This consultation will help interpret the findings and develop recommendations for practice and future research [15].

The objective of this scoping review is to identify and map the available scientific evidence on social skills and emotional regulation in medical students. Specifically, this review aims to:

1. Characterize existing studies on social skills and emotional regulation in medical students in terms of study design, population, interventions, and evaluated outcomes;
2. Identify gaps in current knowledge and areas for future research;
3. Provide a synthesis of available evidence to inform educational practice and the development of medical curricula that address social skills and emotional regulation.

## Methods

The objectives of this methods paper are:

1. To present a detailed and replicable protocol for conducting a scoping review on medical students' mental health, focusing on depression, anxiety, stress, and burnout.
2. To describe the methodological adaptations and innovations implemented to optimize the scoping review process in this specific context.
3. To provide step-by-step guidance for applying this method in scoping reviews on other themes related to mental health in student populations.

### Study design

This study will employ a scoping review model to map the available literature, synthesize the evidence, and identify gaps in this specific area of interest.

### Research question

The research question guiding this scoping review is: What is the scientific evidence on social skills and emotional regulation in medical students? This question was established using the PCC (Population, Concept, Context) strategy, which is widely used to formulate research questions in scoping and systematic reviews. Table 1 presents the PCC.

**Table 1:** PCC Question.

Population	Medical Students
Concept	Social Skills
Context	Emotional Regulation

### Inclusion and exclusion criteria

The inclusion and exclusion criteria were defined based on the research question and the review's objective. Studies on social skills and regulation in medical students, published in English, Portuguese, or Spanish, from January 2019 April 2024 will be included. Primary studies, systematic reviews, meta-analyses, and clinical guidelines will be considered. On the other hand, studies that do not fall within the scope of the review, duplicate publications, letters to the editor, editorials, and opinion articles will be excluded.

### Search strategy

The search strategy has been expanded and refined to ensure a comprehensive and sensitive search across multiple databases. A librarian specializing in digital search strategies has collaborated with the research team to develop the following approach:

1. Database selection: In addition to the previously mentioned databases (EMBASE, ERIC, PubMed, Science Direct, Scopus, and Web of Science), we will also search PsycINFO and CINAHL to capture additional relevant literature from psychology and nursing fields [24].
2. Search terms: The search strategy will use a combination of controlled vocabulary (e.g., MeSH terms, EMTREE terms) and free-text terms. The search terms will be organized into three main concepts: (1) medical students, (2) social skills, and (3) emotional regulation. Synonyms and related terms for each concept will be included to maximize sensitivity.
3. Boolean operators and proximity searches: Boolean operators (AND, OR, NOT) will be used to combine search terms. Proximity operators (e.g., NEAR, ADJ) will be employed where appropriate to capture phrases and related terms within a specified number of words.
4. Filters and limits: Date limits (January 2019 to April 2024) and language restrictions (English, Portuguese, Spanish) will be applied. No other filters will be used to ensure comprehensive coverage.
5. Gray literature search: In addition to database searches, we will conduct a thorough gray literature search [25]. This will include: - Searching relevant conference proceedings (e.g., Association for Medical Education in Europe, International Association for Medical Education) - Examining websites of relevant organizations (e.g., World Health Organization, Association of American Medical Colleges) - Searching dissertation and thesis databases (e.g., ProQuest Dissertations & Theses Global) - Utilizing gray literature-specific search engines (e.g., OpenGrey, Grey Literature Report).
6. Hand searching: Reference lists of included studies and relevant review articles will be hand-searched to identify additional eligible studies.
7. Search documentation: All search strategies will be documented in detail, including the exact search terms, combinations, and results for each database. This documentation will be included as an appendix in the final review to ensure transparency and reproducibility (Table 2).

**Table 2:** Expanded search strategy to PubMed.

Number	Description	Search strategy
#1	Population	“Students, Medical”[Mesh] OR “medical student*”[tiab] OR “medical undergrad*”[tiab] OR “medical education”[tiab]
#2	Concept (Social Skills)	“Social Skills”[Mesh] OR “social skill*”[tiab] OR “interpersonal skill*”[tiab] OR “communication skill*”[tiab] OR “Emotional Intelligence”[Mesh] OR “emotional intelligence”[tiab] OR “social competenc*”[tiab] OR “social abilit*”[tiab]
#3	Context (Emotional Regulation)	“Emotional Regulation”[Mesh] OR “emotion* regulat*”[tiab] OR “affect regulat*”[tiab] OR “mood regulat*”[tiab] OR “emotion* management”[tiab] OR “emotion* control”[tiab] OR “coping strateg*”[tiab]
#4	Combined search	#1 AND #2 AND #3
#5	Date and language limits	#4 AND (“2019/01/01”[Date - Publication]: “2024/04/30”[Date - Publication]) AND (English[lang] OR Portuguese[lang] OR Spanish[lang])

This comprehensive search strategy will ensure that all relevant literature is captured for the scoping review.

## Study selection

The study selection process will be performed by two reviewers independently on the Rayyan platform [26,27]. Initially, titles and abstracts will be screened, followed by full-text reading of potentially relevant studies. Discrepancies will be resolved by consensus or by a third reviewer [28]. The selection process will follow the recommendations of PRISMA (Preferred Reporting Items for Systematic Reviews and Meta-Analyses) and will be presented through a flowchart [29].

## Data extraction

Data will be extracted using a standardized form developed specifically for this review. The form will be pilot-tested on a sample of included studies and refined as necessary. Two reviewers will independently extract data from each included study to ensure accuracy and completeness.

The complete data extraction form is provided in the Appendix. This form has been designed to capture all relevant information as described in the data extraction process. The main categories of information to be extracted include:

1. Study characteristics: Author(s), year of publication, country, study design, sample size;
2. Population details: Age range, gender distribution, year of medical school;
3. Intervention details (if applicable): Type of intervention, duration, frequency, delivery method;
4. Social skills measures: Specific skills assessed, measurement tools used, outcomes reported;
5. Emotional regulation measures: Strategies assessed, measurement tools used, outcomes reported;
6. Key findings: Main results related to social skills and emotional regulation;
7. Implications: Authors' conclusions and recommendations for practice or future research;
8. Quality assessment: Using appropriate tools based on study design (e.g., Mixed Methods Appraisal Tool - MMAT for mixed-methods studies [30].

Any discrepancies in data extraction between reviewers will be resolved through discussion or consultation with a third reviewer. The extracted data will be compiled in a spreadsheet to facilitate analysis and synthesis.

## Data analysis

The data analysis process will employ a mixed-methods approach to provide a comprehensive understanding of the evidence:

1. Quantitative analysis: - Descriptive statistics will be used to summarize study characteristics, population details, and types of interventions or measures used. - If sufficient homogeneous quantitative data are available, meta-analysis will be considered for specific outcomes (e.g., effects of interventions on social skills or emotional regulation scores) [31]. - Subgroup analyses may be conducted based on factors such as year of medical school, type of intervention, or specific social skills/emotional regulation strategies.
2. Qualitative analysis: - Thematic analysis will be employed to identify recurring themes across studies, particularly for qualitative findings related to students' experiences or perceptions of social skills and emotional regulation in medical education. - The thematic analysis will follow the six-step process outlined by Braun and Clarke (2006) [32]: familiarization with the data, generating initial codes, searching for themes, reviewing themes, defining and naming themes, and producing the report.
3. Evidence mapping: - A visual evidence map will be created to illustrate the distribution of studies across different aspects of social skills and emotional regulation in medical students. - This map will help identify clusters of evidence as well as gaps in the current research landscape.
4. Synthesis of findings: - A narrative synthesis will integrate the quantitative and qualitative findings, providing a comprehensive overview of the current state of knowledge on social skills and emotional regulation in medical students. - The synthesis will address each of the review objectives, highlighting key themes, trends, and gaps in the literature.
5. Quality assessment: - The quality of included studies will be critically appraised using appropriate tools based on study design (e.g., MMAT for mixed-methods studies [30], Risk Of Bias In Non-randomized Studies - of Interventions – ROBINS-I for non-randomized interventions [33]). The quality assessment results will be considered when interpreting and reporting the findings of the review.

The identification of research gaps will be conducted through a systematic three-step process:

1. Mapping of themes and subthemes addressed in the included studies.
2. Analysis of the frequency and depth with which each theme/subtheme is addressed.
3. Identification of underexplored areas or inconsistencies in results across studies. This process will be conducted independently by two reviewers, with discussion to resolve any discrepancies.

This comprehensive analysis approach will ensure a thorough examination of the available evidence, providing valuable insights for medical education practice and future research directions.

## Discussion

The results of this scoping review will provide a comprehensive overview of the available scientific evidence on social skills and emotional regulation in medical students. It is expected that the findings can inform the development of medical curricula that address these essential skills for student well-being and performance. Furthermore, identifying gaps in current knowledge may guide future research in this area. The expanded search strategy and rigorous analysis process will contribute to the strength and comprehensiveness of this review. By including a wide range of databases and gray literature sources, we aim to capture a broad spectrum of evidence on social skills and emotional regulation in medical education. The mixed-methods analysis approach will allow for a nuanced understanding of both quantitative outcomes and qualitative experiences related to these skills. The findings of this review may have significant implications for medical education practice. By synthesizing evidence on effective interventions and strategies for enhancing social skills and emotional regulation, educators can make informed decisions about curriculum design and student support services. Additionally, the identification of gaps in the current research landscape will help guide future studies, potentially leading to more targeted and effective interventions for improving medical students' well-being and professional development. A potential limitation of this review is the heterogeneity of the included studies, which may hinder the synthesis of results. However, the scoping review approach is suitable for dealing with this diversity and providing an overview of the current state of knowledge. To address this limitation, we will carefully categorize and analyze studies based on their methodological approaches, populations, and specific outcomes. This stratified analysis will allow us to identify patterns and trends across different types of studies, even in the presence of heterogeneity. Another consideration is the rapid pace of change in medical education, particularly in light of recent global events such as the COVID-19 pandemic. To account for this, we will pay special attention to recent studies and emerging trends in social skills and emotional regulation interventions, highlighting any shifts in approach or focus that may have occurred in response to changing educational environments.

## Conclusion

This scoping review will map the scientific evidence on social skills and emotional regulation in medical students. Through a comprehensive search strategy, rigorous data extraction, and mixed-methods analysis, this review will provide a nuanced understanding of the current state of knowledge in this important area. The results may inform educational practice and identify areas for future research, contributing to the development of strategies that promote the well-being and performance of medical students. By addressing social skills and emotional regulation in medical curricula, educators can better prepare future physicians for the complex interpersonal and emotional demands of medical practice.

## Protocol validation

Not applicable.

## Limitations

Not applicable.

## CRediT authorship contribution statement

**Thaysa Castro Molina:** Conceptualization, Methodology, Writing – original draft, Writing – review & editing, Visualization. **William Donegá Martinez:** Writing – review & editing. **Vinicíus Araújo Pereira:** Methodology, Writing – review & editing. **Alba Regina de Abreu Lima:** Writing – review & editing. **Vânia Maria Sabadoto Brienze:** Writing – review & editing. **Emerson Roberto dos Santos:** Writing – review & editing. **Valdir Carlos Severino Júnior:** Writing – review & editing. **Camila Borge:** Writing – review & editing. **Júlio César André:** Writing – review & editing.

## Declaration of competing interest

The authors declare that they have no known competing financial interests or personal relationships that could have appeared to influence the work reported in this paper.

## Data Availability

Data will be made available on request.

## Appendix Supplementary materials

Image, application 1
