# Supplementary material for: Toward evidence-informed medical curricula: A scoping review protocol on social skills and emotional regulation
Source: MethodsX. 2025 Jan 11;14:103161. doi: 10.1016/j.mex.2025.103161 (PMC11787439; doi:10.1016/j.mex.2025.103161)
Supplement: Supplementary file 1 [file mmc1.docx]

Appendix: Data extraction form.

Study Identification

- Authors:
- Year of publication:
- Country:
- Study ID:

Study Characteristics

- Objective/Aim:
- Study design:
  - Randomized controlled trial
  - Non-randomized controlled trial
  - Cohort study
  - Case-control study
  - Cross-sectional study
  - Qualitative study
  - Mixed-methods study
  - Systematic review
  - Meta-analysis
  - Clinical guideline
  - Other (specify):
- Study duration:

Population Characteristics

- Sample size:
- Age (mean, SD, range):
- Gender (% male/female):
- Year of study:
- Specialty (if applicable):
- Inclusion criteria:
- Exclusion criteria:

Intervention (if applicable)

- Type of intervention:
- Duration of intervention:
- Frequency of intervention:
- Setting:
- Delivered by:

Comparator (if applicable)

- Type of comparator:
- Duration of comparator:
- Frequency of comparator:
- Setting:
- Delivered by:

Outcomes

- Primary outcomes:
  - Outcome 1:
    - Definition:
    - Measurement tool/method:
    - Time points measured:
  - Outcome 2:
    - Definition:
    - Measurement tool/method:
    - Time points measured:
- Secondary outcomes:
  - Outcome 1:
    - Definition:
    - Measurement tool/method:
    - Time points measured:
  - Outcome 2:
    - Definition:
    - Measurement tool/method:
    - Time points measured:

Results

- Main findings:
- Effect sizes (if reported):
- Confidence intervals (if reported):
- p-values (if reported):

Conclusions

- Key conclusions:
- Implications for practice:
- Implications for research:

Limitations

- Limitations reported by authors:
- Limitations identified by reviewers:

Quality Assessment (if applicable)

- Tool used:
- Rating:

Additional Notes

- Funding sources:
- Conflicts of interest:
- Other relevant information:
